# Spray vaccination with a safe and bivalent H9N2 recombinant chimeric NDV vector vaccine elicits complete protection against NDV and H9N2 AIV challenge

**DOI:** 10.1186/s13567-025-01448-5

**Published:** 2025-01-31

**Authors:** Xiaoquan Wang, Jing Dai, Wenhao Yang, Yao Yao, Jin Zhang, Kaituo Liu, Xiaolong Lu, Ruyi Gao, Yu Chen, Jiao Hu, Min Gu, Shunlin Hu, Xiufan Liu, Xiaowen Liu

**Affiliations:** 1 Key Laboratory of Avian Bioproducts Development, Ministry of Agriculture and Rural Affairs, Yangzhou University, Yangzhou, 225000 China; 2 Jiangsu Co-Innovation Center for Prevention and Control of Important Animal Infectious Diseases and Zoonosis, Yangzhou University, Yangzhou, 225000 China; 3 Jiangsu Key Laboratory of Zoonosis, Yangzhou University, Yangzhou, 225000 China; 4 Joint International Research Laboratory of Agriculture and Agri-Product Safety of Ministry of Education of China, Yangzhou University, Yangzhou, 225000 China

**Keywords:** H9N2 subtype avian influenza virus, Newcastle disease virus, chicken, vaccine, spray vaccination

## Abstract

Newcastle disease virus (NDV) and H9N2 avian influenza virus (AIV) represent significant pathogenic risks to the poultry industry, leading to considerable economic losses. Vaccination is a widely used preventive measure against these pathogens, yet the lack of a live bivalent vaccine targeting NDV and H9N2 AIV imposes a heavy vaccination burden. Previously, we constructed a genotype-matched chimeric NDV vector, LX-OAI4S, in which the genotype I NDV backbone was replaced with the ectodomain of haemagglutinin-neuraminidase (HN) and modified using the attenuated F gene from the genotype VII vaccine strain A-VII. Based on the LX-OAI4S vector, we successfully generated three H9N2 recombinant viruses: LX-OAI4S-NPU-HA, LX-OAI4S-MU-HA, and LX-OAI4S-HNU-HA. These recombinants incorporated the H9N2 HA gene, flanked by untranslated regions (UTRs) from the NP, M, or HN gene of the NDV LX strain, inserted between the P and M genes of LX-OAI4S. The vaccine candidate LX-OAI4S-NPU-HA induced a more robust immune response in chickens against H9N2 AIV and NDV than the other two recombinants. This response effectively protects against virus shedding and lethal virus challenge. Furthermore, spray vaccination with LX-OAI4S-NPU-HA showed protective efficacy against H9N2 AIV and NDV. This study offers a promising strategy for comprehensive protection in regions threatened by H9N2 AIV and NDV.

**Supplementary Information:**

The online version contains supplementary material available at 10.1186/s13567-025-01448-5.

## Introduction

Avian influenza viruses (AIVs) belong to the genus *Influenza A virus* of the *Orthomyxoviridae* family. AIVs encompass various subtypes, such as H5N1, H7N9, and H9N2, distinguished by different combinations of hemagglutinin (HA) and neuraminidase (NA). According to epidemiological studies, H9N2 is the most prevalent subtype in China and globally [1, 2]. AIVs are divided into highly pathogenic avian influenza virus (HPAIV) and low pathogenic avian influenza virus (LPAIV) based on their pathogenicity [3]. The H9N2 subtype, classified as LPAIV, typically causes no significant clinical signs or death in chickens when infection occurs alone. However, co-infection with other pathogens can precipitate severe clinical symptoms and potentially lead to fatalities [4–7].

Newcastle disease (ND), caused by the Newcastle disease virus (NDV), damages the poultry population and causes substantial economic losses within the global poultry industry [8]. Although ND is somewhat controlled in China, its recurrence in neighbouring regions and the potential for invasion poses a considerable threat to the control efforts in China. These evidences demonstrate the necessity of preventing and controlling H9N2 AI (avian influenza) and ND. The current preventive measure against H9N2 AI and ND in China is vaccination, which mainly consists of inactivated whole virus vaccines [9, 10]. The vaccine swiftly boosts humoral immunity, yielding high antibody levels post-immunisation, thus demonstrating efficacy in the early stages of outbreaks.

However, antigenic drift causes a vaccine-strain mismatch with circulating strains, undermining the protective efficacy of traditional inactivated vaccines [11]. Furthermore, the inactivated whole virus vaccine prompts robust humoral immunity but fails to prevent reinfection and halt virus shedding in vaccinated chickens [12]. Therefore, there is an urgent need for a new and effective vaccine to prevent and control H9N2 AI and ND in poultry. Nonetheless, advances in molecular biology have expanded the use of viral vectors, such as NDV, avian pox virus, and turkey herpesvirus, for recombinant avian influenza vaccines [13–15]. NDV, long used as a vaccine vector, now includes recombinant ND vaccines expressing exogenous genes from pathogens like AIV, infectious bronchitis virus, and the recent emergence of SARS-CoV-2 [15–17].

The untranslated regions (UTRs) of NDVs are crucial for exogenous gene expression. Previous studies have shown that merging M and F gene UTRs with exogenous protein flanks boosts foreign protein expression [18]. In our prior research, we developed a hybrid vector, LX-OAI4S, by fusing genotype I and VIId NDV. We used the LX backbone, replacing its glycoprotein with HN ectodomain and modified F proteins from A-VII and the ND vaccine issued in China. The LX-OAI4S vaccine induces strong mucosal immunity and is safe for spray administration.

Here, we have created three chimeric NDVs by inserting the H9N2 AIV HA gene into the LX-OAI4S backbone, with and without NP, M, and HN UTRs. The immunogenicity and protective efficacy of the LX-OAI4S-NPU-HA variant were evaluated in chickens through intraocular nasal or spray vaccination. The results demonstrated immunity against both NDV and H9N2 AIV. This study opens up the opportunity to apply this vaccine platform to develop further a bivalent vaccine against H9N2 subtype AIVs and NDV infection.

## Materials and methods

### Cells and viruses

The BHK-21 cell clone BSR T7/5 stably expressing the phage T7 RNA polymerase was gifted from Prof. Zhigao Bu (Harbin Veterinary Research Institute, Chinese Academy of Agricultural Sciences, China). The chicken embryonic fibroblasts (CEF) were grown and maintained in Dulbecco’s modified Eagle’s Medium (DMEM) supplemented with 10% foetal bovine serum, respectively, at 37 °C with 5% CO_2_ atmosphere. Specific pathogen-free (SPF) chicken embryos were supplied from Zhejiang Lihua Agricultural Technology Co., Ltd. (China). The H9N2 AIV strain A/Chicken/Anhui/AH463/2017 (AH463) (GenBank ID: ON248014.1) and genotype VII virulent NDV strain JS02/06 (GenBank ID: EU044805.1) were stored in our laboratory.

### Plasmid construction

The HA gene of the H9N2 virus strain AH463 was used as the exogenous antigen gene, amplified with the primers. The UTR sequences, flanked by the homology sequence of HA and vector, were generated by annealed oligo primers (Additional file 1). Next, the HA gene flanked by the GS, GE, and UTR sequences of each LX-OAI4S gene was amplified using an overlap polymerase chain reaction (PCR). We linearised the pLX-OAI4S plasmid by restricting endonucleases SacII and AvrII. The fragments from the insertion sites (the P and M gene junction) to SacII or AvrII were amplified separately by PCR using the LX plasmid as a template and subsequently linked to the above PCR products using overlap PCR.

**Figure 1:**
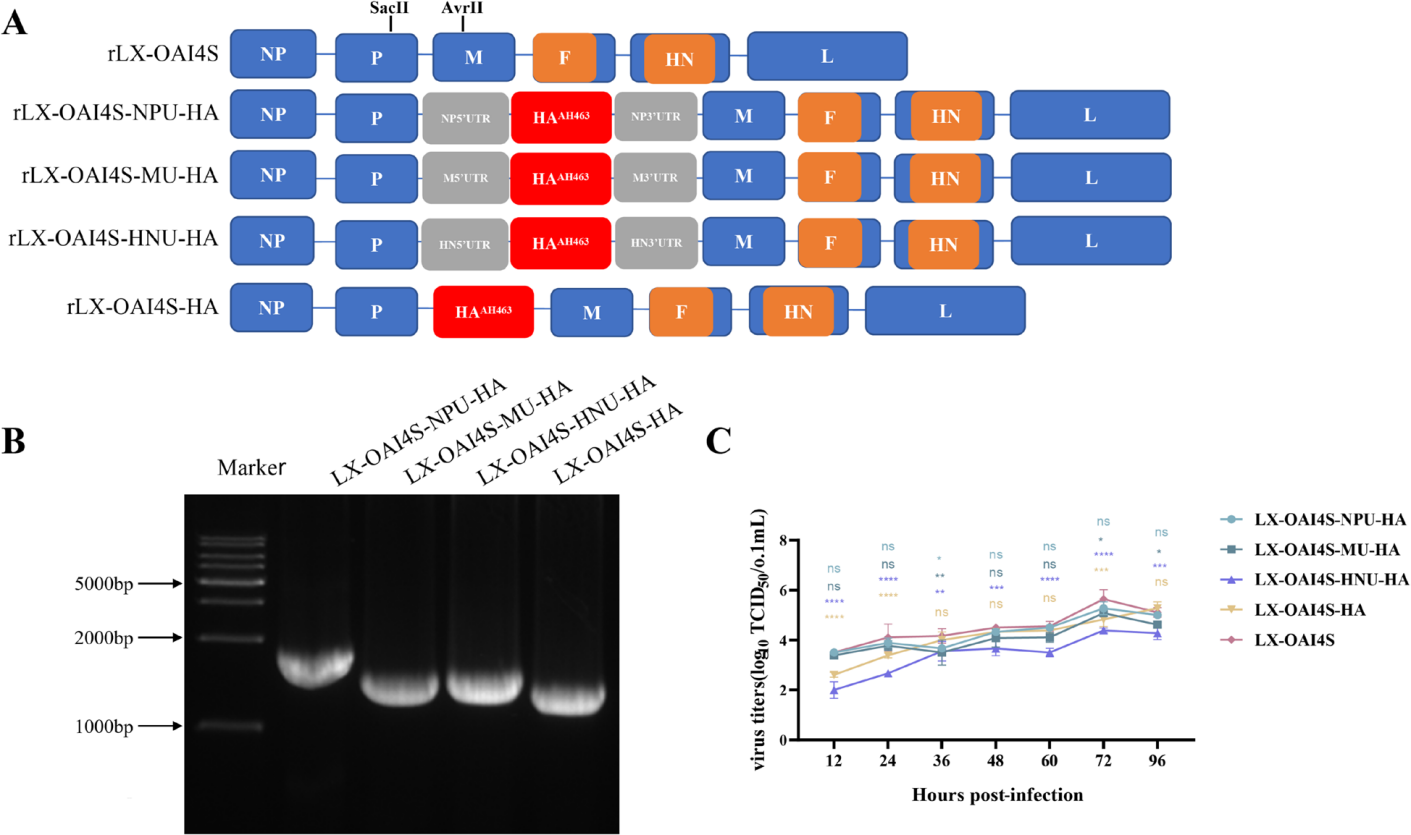
**Construction of recombinant NDV expressing H9N2 HA gene. A** Schematic diagram of construction of recombinant NDV expressing H9N2 HA gene. H9N2-HA gene, flanked with UTR sequences of NP, M, and HN gene, was inserted between the P and M genes of rLX-OAI4S. rLX-OAI4S-HA did not contain UTR. Restriction endonucleases: SacII and AvrII. **B** Identification of HA gene of recombinant NDV by RT-PCR. **C** Growth curves of rLX-OAI4S/HAs and rLX-OAI4S. *P < 0.05; **P < 0.01; ***P < 0.001; ****P < 0.0001; ns, no significant difference.

The final overlapping PCR products were inserted into the vector through the SacII and AvrII sites using the pEASY^®^-Basic Seamless Cloning and Assembly Kit (Transgen, Beijing, China). The recombinant plasmids of LX-OAI4S-NPU-HA, LX-OAI4S-MU-HA, LX-OAI4S-HNU-HA, and LX-OIA4S-HA were then constructed (Figure 1A). Three supporting plasmids, pCIZJ1NP, pCIZJ1P, and pCIZJ1L, expressing the NP, P, and L genes, were constructed by our lab.

### Generation of the recombinant NDV expressing H9 HA

The recombinant viruses were generated using reverse genetics techniques, following the previously described methods. In brief, BSR T7/5 cells were seeded in 6-well plates at a density of 6 × 10^5^ cells/well before transfection. The cells were initially inoculated with the modified vaccinia Ankara virus at a multiplicity of infection (MOI) of 1. They were then transfected with a mixture containing the full-length cDNA and three supporting plasmids using the X-tremeGENE HP DNA Transfection Reagent (Roche, Germany). The cells and supernatant mixture were collected at 96 hours post-transfection (hpt) and inoculated into 9-day-old SPF chicken embryos. The allantoic fluids were then collected for haemagglutination (HA) assay to identify the rescued virus.

### Characterisation of the recombinant NDV expressing H9 HA

The recombinant viruses were passaged up to the fifth generation in chicken embryos, and their HA titres were evaluated. A 50% egg infectious dose (EID_50_) test was performed by serial tenfold dilution of recombinant virus and inoculating the dilution into 9-day-old SPF chicken embryos. The tissue culture infectious dose (TCID_50_) test was conducted by inoculating CEF cells with a serial tenfold dilution of the recombinant virus.

The EID_50_ and TCID_50_ values of the viruses were determined according to the Reed-Muench method. The pathogenicity of the recombinant viruses was established by the mean death time (MDT) and the intracerebral pathogenicity index (ICPI) according to the recommendations of the World Organization for Animal Health (WOAH) [19]. We then compared the growth kinetics of the four recombinant viruses against LX-OAI4S in CEF according to TCID_50_. To assess the stability of these recombinant viruses, we conducted sequencing following the serial passages of the viruses. Briefly, the recombinant virus was subjected to 20 passages in SPF chicken embryos, with RNA extraction from allantoic fluid at 5-generation intervals for reverse transcription. Subsequent PCR and sequencing targeted the inserted HA gene and the virus’s native F gene.

### Western blot and indirect immunofluorescence assay

To verify the expression level of the H9N2 HA protein, CEF were infected with each virus at 1 MOI. The total protein from cell extraction was boiled with the loading buffer (CWBio, China) for 10 min. The extracted proteins were separated on SDS–polyacrylamide gels, transferred to polyvinylidene fluoride membranes, blocked with blocking buffer (NcmBlot, China), and probed with monoclonal chicken anti-H9N2-HA and chicken anti-NDV-NP antibodies (prepared by our lab) overnight at 4 °C. This process was followed by incubation with horseradish peroxidase-conjugated goat anti-chicken IgY (H + L) antibodies (Abcam, USA).

We obtained the protein bands with an electrochemiluminescence detection system (Tanon, China). β-actin (Sigma Chemical, USA) was used as an internal control. The anti-H9N2-HA antibody was likewise selected as an indirect immunofluorescence assay (IFA). Goat anti-chicken IgY (H + L) FITC (Thermo Fisher Scientific, USA) was used as the secondary antibody.

### Animal vaccination via intranasal and intraocular route

For this study, we purchased three-week-old SPF chickens from Zhejiang Lihua Agricultural Technology Co., Ltd. (China) and randomly divided them into five groups. Four groups of chickens were vaccinated by the intranasal and intraocular routes with 10^6^ EID_50_/0.1 mL of LX-OAI4S-NPU-HA, LX-OAI4S-MU-HA, LX-OIA4S-HA, and LX-OAI4S, respectively. Chickens in the control group were inoculated similarly with PBS.

Weekly blood samples were taken through the 21 days post-immunisation (dpi), and serum HI antibody levels against H9 AIV and NDV were assessed. Subsequently, each group of chickens was randomly divided into two subgroups. One subgroup was challenged with H9N2 AIV, while the other was challenged with NDV. Both challenges were administered via intranasal and intraocular routes, with a 10^6^ EID_50_/0.1 mL dosage. The lung, trachea, spleen, thymus, and duodenum tissue samples were preserved in 10% phosphate-buffered formalin at 5 days post-challenge (dpc). We then processed samples for paraffin embedding by cutting them into 5 µm sections and staining them with haematoxylin and eosin (H&E). The lung, trachea, larynx, duodenum, brain, and spleen samples were collected at 3 and 5 dpc for viral detection and titrated by quantitative reverse transcription polymerase chain reaction (RT-qPCR). The primers used are shown in Additional file 2. Finally, the oral and cloacal swabs were collected from all the chickens for virus shedding detection using SPF embryonated chicken eggs at 3, 5, and 7 dpc.

### Animal vaccination via spray administration route

One-day-old SPF Babcock chickens were immunised with LX-OAI4S-NPU-HA and LX-OAI4S by spray administration. In brief, we vaccinated three groups of one-day-old chicks with 2 × 10^6^ EID_50_/0.1 mL or 10 × 10^6^ EID_50_/0.1 mL of recombinant virus or PBS as a control. We used a vaccine spraying machine (Xin Sheng Yuan, China) with a droplet size of 140–180 μm and a spray volume of 16 mL/100 chickens. After the spraying, the chicks were required to remain in the spray box for over 3 s.

Weekly blood samples were taken through 21 dpi, and the serum levels of HI antibodies against H9 AIV and NDV were assessed. The LX-OAI4S-NPU-HA group was then randomly divided into two subgroups. One subgroup was challenged with H9N2 AIV, while the other was challenged with NDV. Additionally, the LX-OAI4S group was challenged with NDV. Both challenges were administered through intranasal and intraocular routes, with a 10^6^ EID_50_/0.1 mL dosage. Oral and cloacal swabs were taken from all chickens to detect virus shedding using SPF embryonated chicken eggs on days 3, 5, and 7 dpc.

### Statistical analysis

Statistical analysis of variance was performed by one-way Analysis of Variance (ANOVA) using GraphPad Prism 8.0.2 (GraphPad Software, USA). Differences were considered statistically significant at *P < 0.05; **P < 0.01; ***P < 0.001; ****P < 0.0001.

## Results

### Biological properties of four recombinant viruses

Four recombinant NDV were obtained through reverse genetics techniques: rLX-OAI4S-NPU-HA, rLX-OAI4S-MU-HA, rLX-OAI4S-HNU-HA, and rLX-OIA4S-HA (Figure 1A). RT-PCR and sequencing identification of the exogenous HA gene showed no mutation (Figure 1B).

Subsequently, we measured the replication efficiency and virulence of these recombinant viruses. Their replication kinetics in CEF cells were consistent with those of the parental virus, except for LX-OAI4S-HNU-HA, which exhibited a distinct replication pattern (Figure 1C). The four recombinant viruses exhibited replication efficiency in chicken embryos fundamentally identical to the parent virus, with 8log_2_ or higher for the HA titre. Notably, the EID_50_ values of the rescued viruses followed a similar trend to the HA titres.

The MDT for all recombinant viruses extended beyond 120 h. Furthermore, the ICPI values for all recombinant viruses were less than 0.7, indicating reduced virulence (Table 1). Moreover, the sequence analysis of the recombinant viruses following serial passages revealed no mutations, confirming their genetic stability (Additional file 3).

**Table 1:** **Biological characteristics of rLX-OAI4S/HAs and rLX-OAI4S**

Strain	HA (log_2_)	EID_50_/0.1 mL	MDT	TCID_50_/0.1 mL	ICPI
LX-OAI4S-NPU-HA	9	8.83	> 120 h	6.83	0
LX-OAI4S-MU-HA	8	9.17	> 120 h	6.83	0
LX-OAI4S-HNU-HA	8	9.17	> 120 h	6.17	0
LX-OAI4S-HA	8	8.67	> 120 h	6.83	0
LX-OAI4S	9	9	> 120 h	7.50	0

### Highly efficient expression of the HA protein in recombinant chimeric NDVs

**Figure 2:**
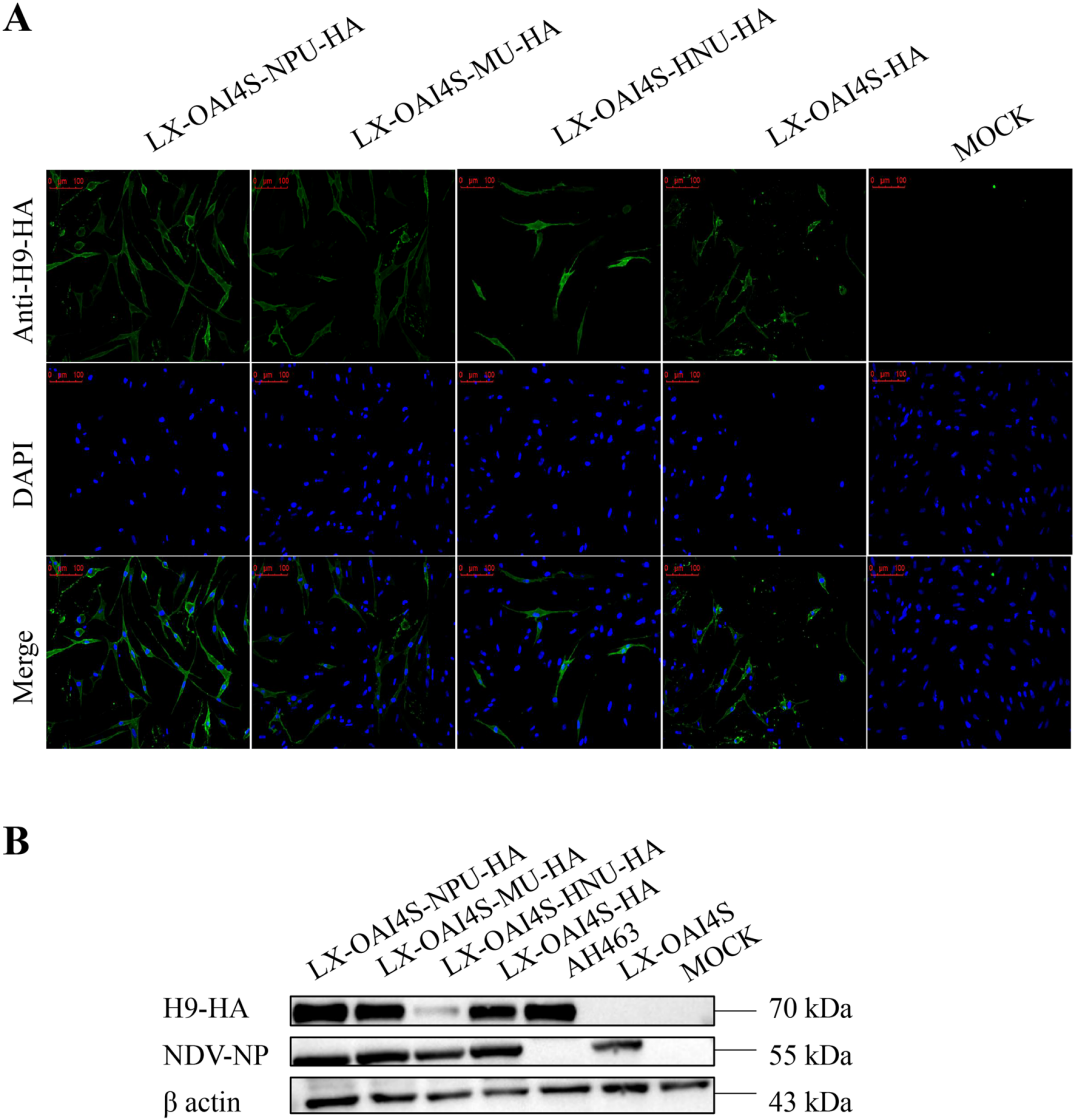
**Characterization of HA protein expression of recombinant viruses. A** Expression levels of H9-HA protein of rLX-OAI4S/HAs in CEF by IFA. Scale bar: 100 μm. **B** Expression levels of H9-HA protein and NDV-NP protein of recombinant viruses by Western blot analysis in CEF. Equal protein loading was confirmed with the β-actin antibody.

The expression of HA proteins of the four recombinant viruses was determined by IFA (Figure 2A) and western blot (Figure 2B) assays. The results indicated that all the exogenous H9-HA proteins from the four recombinant viruses could be expressed in the NDV vector. The rLX-OAI4S-NPU-HA recombinant virus exhibited the highest HA protein expression, surpassing MUTR, with HNUTR showing the lowest expression, even falling below the level of the UTR-less recombinant virus. In addition, the expression of NDV-NP protein in recombinant viruses was similar, with no significant deviation from the NP protein expression level of the parental virus.

### Intranasal and intraocular vaccination with recombinant chimeric NDV provides protection against NDV and H9N2 AIV

In our study, the recombinant chimeric virus rLX-OAI4S-HNU-HA exhibited reduced protein expression compared to the other three recombinant viruses. It also displayed diminished growth kinetics in cell culture relative to the vector viruses. Therefore, we employed the recombinant viruses to assess their immune protection efficacy against H9N2 AIV and NDV, excluding rLX-OAI4S-HNU-HA from this evaluation.

**Figure 3:**
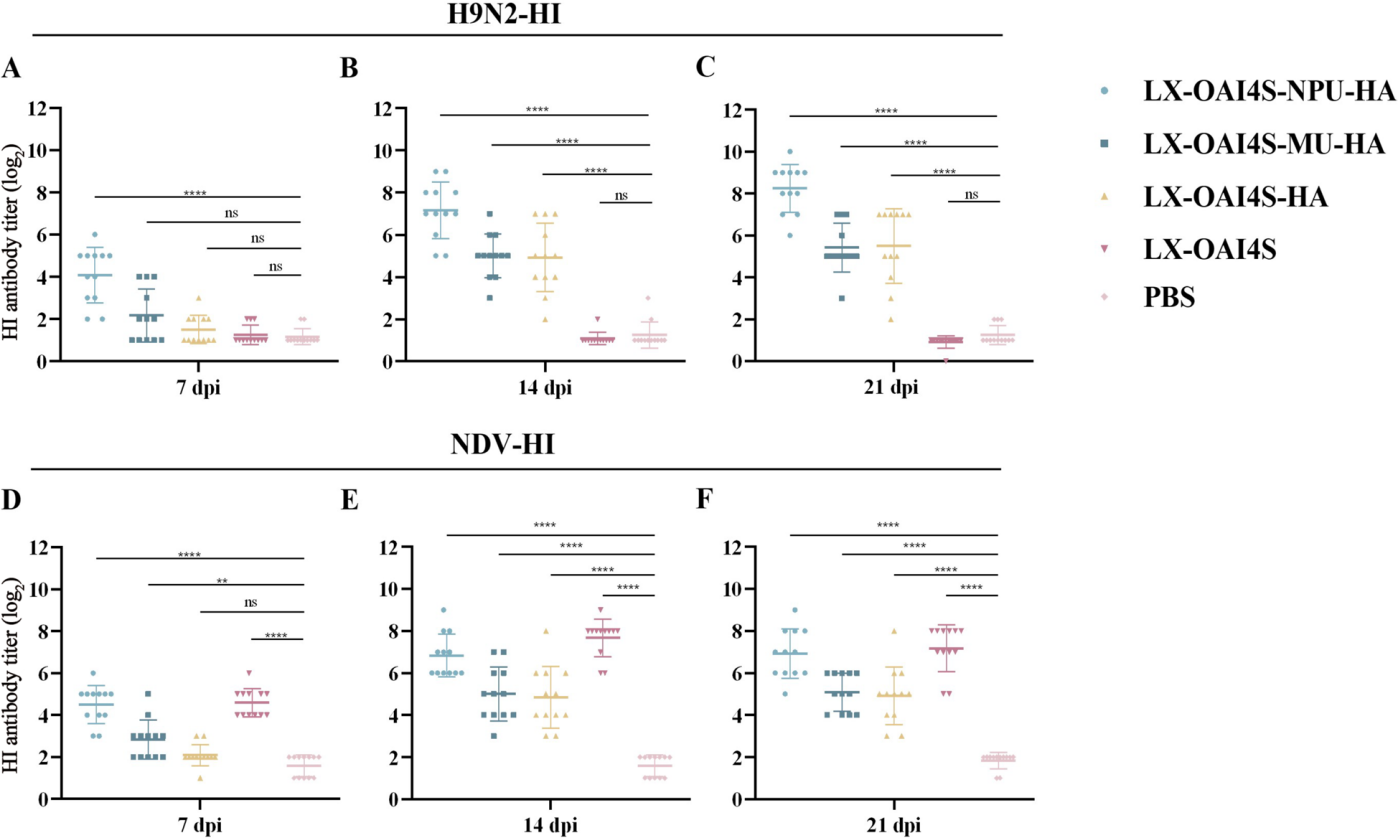
**HI antibody titers of SPF chickens immunized through the intranasal and intraocular route. A**–**C** LX-OAI4S-NPU-HA, LX-OAI4S-MU-HA, LX-OAI4S-HA, LX-OAI4S-HA, LX-OAI4S and PBS were immunized with 10^6^EID_50_ for HI antibody titers targetingH9N2 in 3-week-old SPF chickens at 7, 14, and 21 days, respectively. **D**–**F** LX-OAI4S-NPU-HA, LX-OAI4S-MU-HA, LX-OAI4S-HA, LX-OAI4S-HA, LX-OAI4S and PBS were immunized with 10^6^ EID_50_ for HI antibody titers targeting NDV in 3-week-old SPF chickens at 7, 14, and 21 days, respectively. The *P*-values are shown for comparing PBS and rLX-OAI4S/HAs and the rLX-OAI4S vaccine group. *P < 0.05; **P < 0.01; ***P < 0.001; ****P < 0.0001; ns, no significant difference.

Following vaccination, the vaccinated group and the PBS-inoculated controls showed no clinical symptoms and maintained regular weight gain during the vaccination phase. All recombinant viruses successfully induced HI antibody titres against H9N2 AIV and NDV (Figure 3). The rLX-OAI4S-NPU-HA (NPUTR) recombinant virus exhibited the highest antibody titres, with an average HI antibody titre of 8.5log_2_ against H9N2 AIV and an average HI antibody titre of 6.9log_2_ against NDV at 21 dpi. Subsequently, the challenge of H9N2 and NDV tests were performed at 21 dpi, and all immunised groups showed no clinical symptoms or mortality.

**Figure 4:**
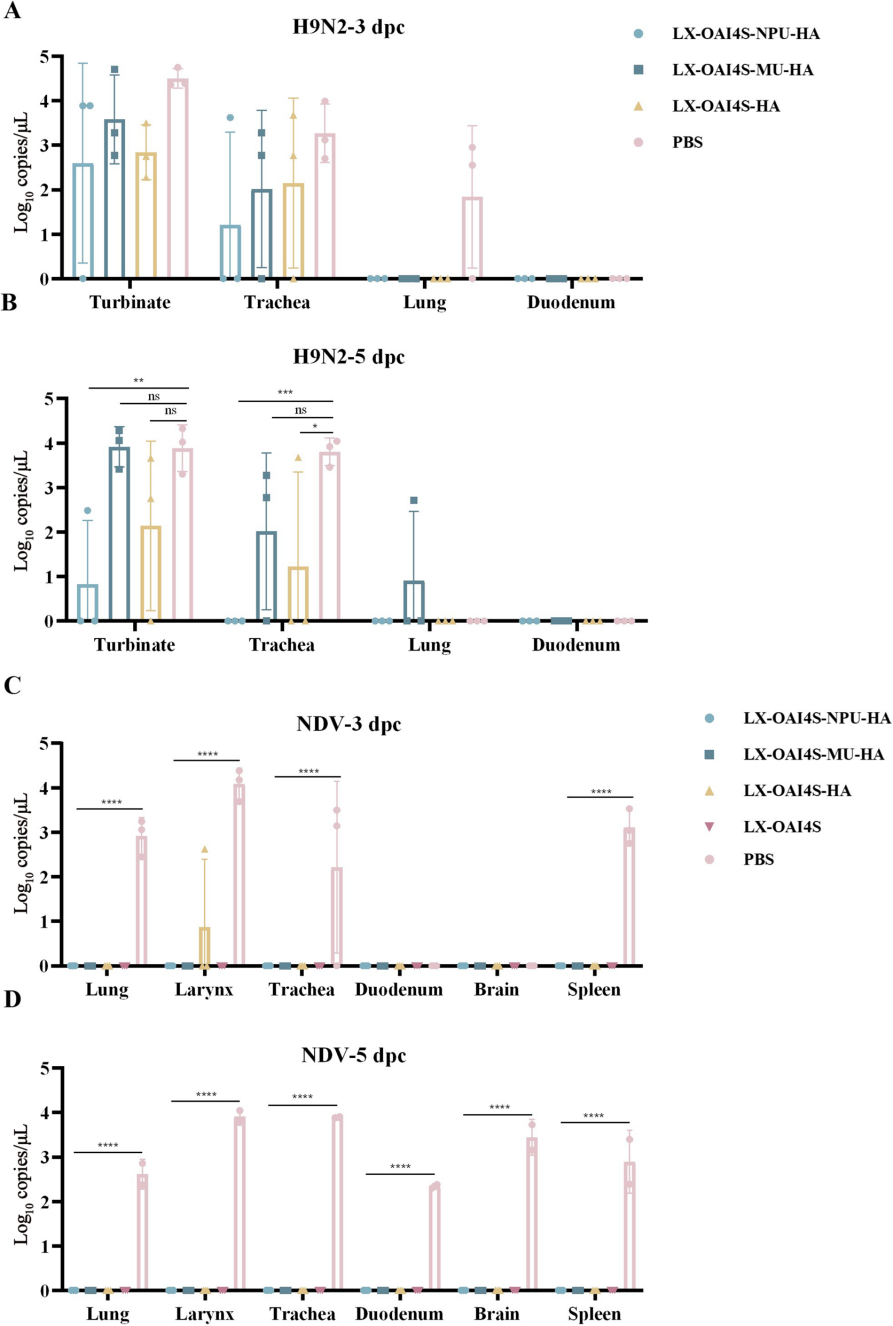
**The replication levels of viruses in vivo following immunization.** The viral loads in each turbinate, trachea, lung, and duodenum were detected by RT-qPCR at 3 (**A**) and 5 (**B**) dpc. The viral loads in each lung, trachea, larynx, duodenum, brain, and spleen were detected by RT-qPCR at 3 (**C**) and 5 (**D**) dpc. *P < 0.05; **P < 0.01; ***P < 0.001; ****P < 0.0001; ns, no significant difference.

**Figure 5:**
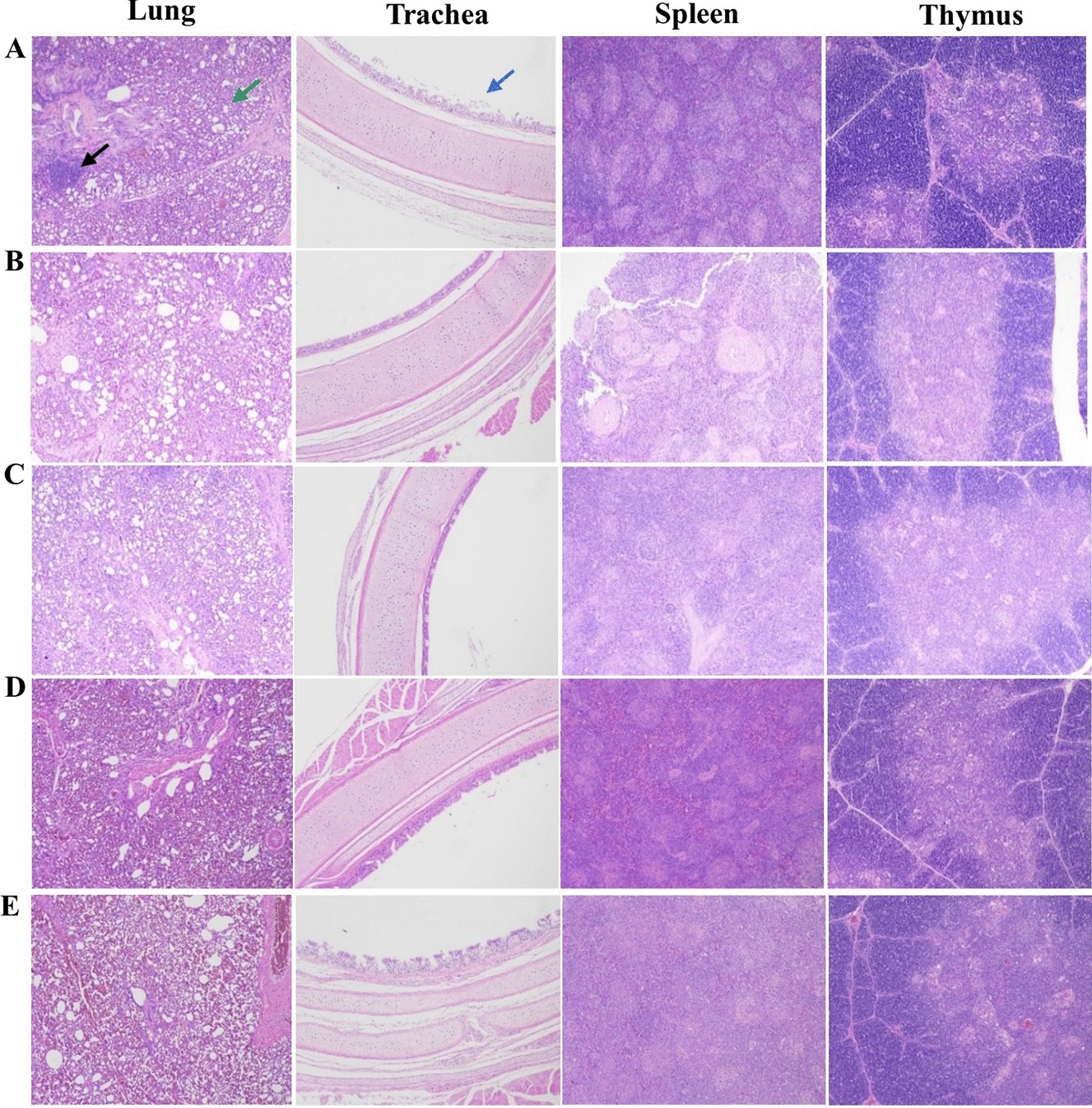
**Visceral pathological changes of each immunization group following H9N2 AIV challenge. A** PBS group; **B** LX-OAI4S-NPU-HA group; **C** LX-OAI4S-MU-HA group; **D** LX-OAI4S-HA group; **E** Mock group. Black arrow: focal infiltration of inflammatory cells; Green arrow: lumen enlargement; Blue arrow: mucosal epithelium has more cilia shedding. Observation was done under light microscope (100 ×).

**Figure 6:**
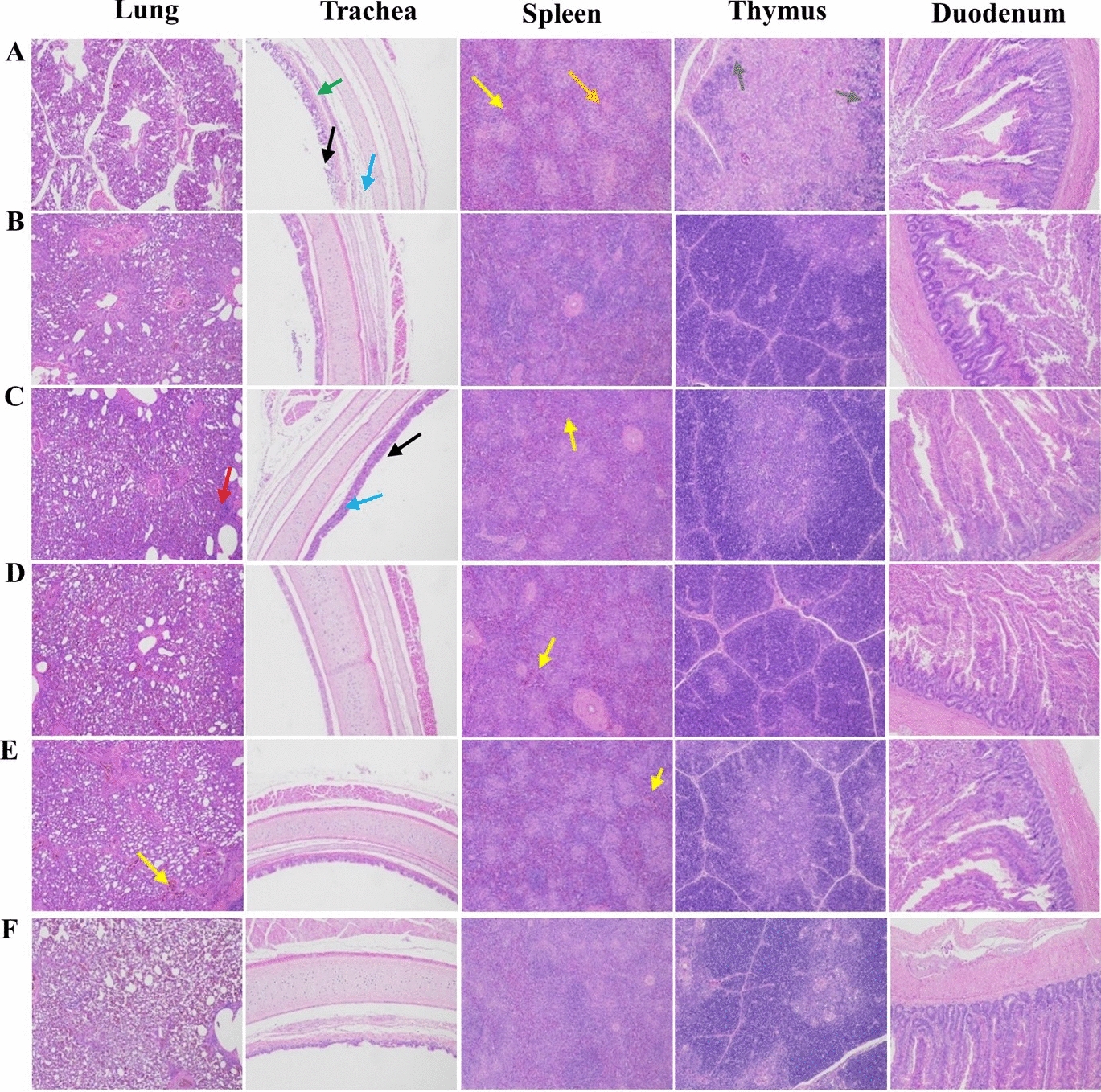
**Visceral pathological changes of each immunization group following NDV challenge. A** PBS group; **B** LX-OAI4S-NPU-HA group; **C** LX-OAI4S-MU-HA group; **D** LX-OAI4S-HA group; **E** LX-OAI4S group; **F** Mock group. Red Arrow: Inflammatory cell infiltration; Yellow arrow: congestion; Black arrow: mucosal epithelial cilia shed; Green arrow: Loose arrangement of connective tissue in lamina propria; Blue arrow: lymphocyte infiltration; Orange arrow: cell necrosis, nucleus fragmentation and dissolution, showing eosinophilic homogeneous substance; Gray arrow: the boundary between cortex and medulla is unclear. Observation was done under light microscope (100 ×).

Furthermore, the NPUTR group had effectively suppressed viral shedding of H9N2 AIV and NDV in both oral and cloacal samples at 5 and 7 dpc, with only a single instance of virus shedding observed in one chicken at 3 dpc following H9N2 infection (Tables 2 and 3). The NPUTR group reduced the replication of both NDV and H9N2 in several organs at 5 dpc, which was significantly different from the PBS group (Figure 4). Additionally, pathological damage was observed in almost all organs collected from the PBS control group after NDV or H9N2 challenge, while the vaccine group significantly inhibited the appearance of organ lesions (Figures 5 and 6).

**Table 2:** **Virus shedding of chickens challenged with H9N2 AIV**

Groups	3 dpc		5 dpc		7 dpc	
	Oral	Cloacal	Oral	Cloacal	Oral	Cloacal
LX-OAI4S-NPU-HA	1/6	0/6	0/6	0/6	0/6	0/6
LX-OAI4S-MU-HA	5/6	0/6	6/6	0/6	1/6	0/6
LX-OAI4S-HA	6/6	2/6	5/6	0/6	0/6	0/6
LX-OAI4S	6/6	4/6	6/6	3/6	0/6	0/6
PBS	6/6	1/6	6/6	2/6	0/6	0/6

**Table 3:** **Virus shedding of chickens challenged with NDV**

Groups	3 dpc		5 dpc		7 dpc	
	Oral	Cloacal	Oral	Cloacal	Oral	Cloacal
LX-OAI4S-NPU-HA	0/6	0/6	0/6	0/6	0/6	0/6
LX-OAI4S-MU-HA	1/6	0/6	0/6	3/6	0/6	3/6
LX-OAI4S-HA	2/6	2/6	3/6	0/6	0/6	2/6
LX-OAI4S	0/6	0/6	0/6	0/6	0/6	0/6
PBS	6/6	6/6	N/A^a^	N/A	N/A	N/A

^a^NA: not applicable due to death of chickens.

### Spray vaccination recombinant NDV provides protection against NDV and H9N2 AIV

After our preliminary experiments, we established that NPUTR enhanced the immunogenicity of LX-OAI4S-NPU-HA, providing more efficient protection against both H9N2 AI and ND. Therefore, we further investigated the protective effect induced by NPUTR after spray administration.

**Figure 7:**
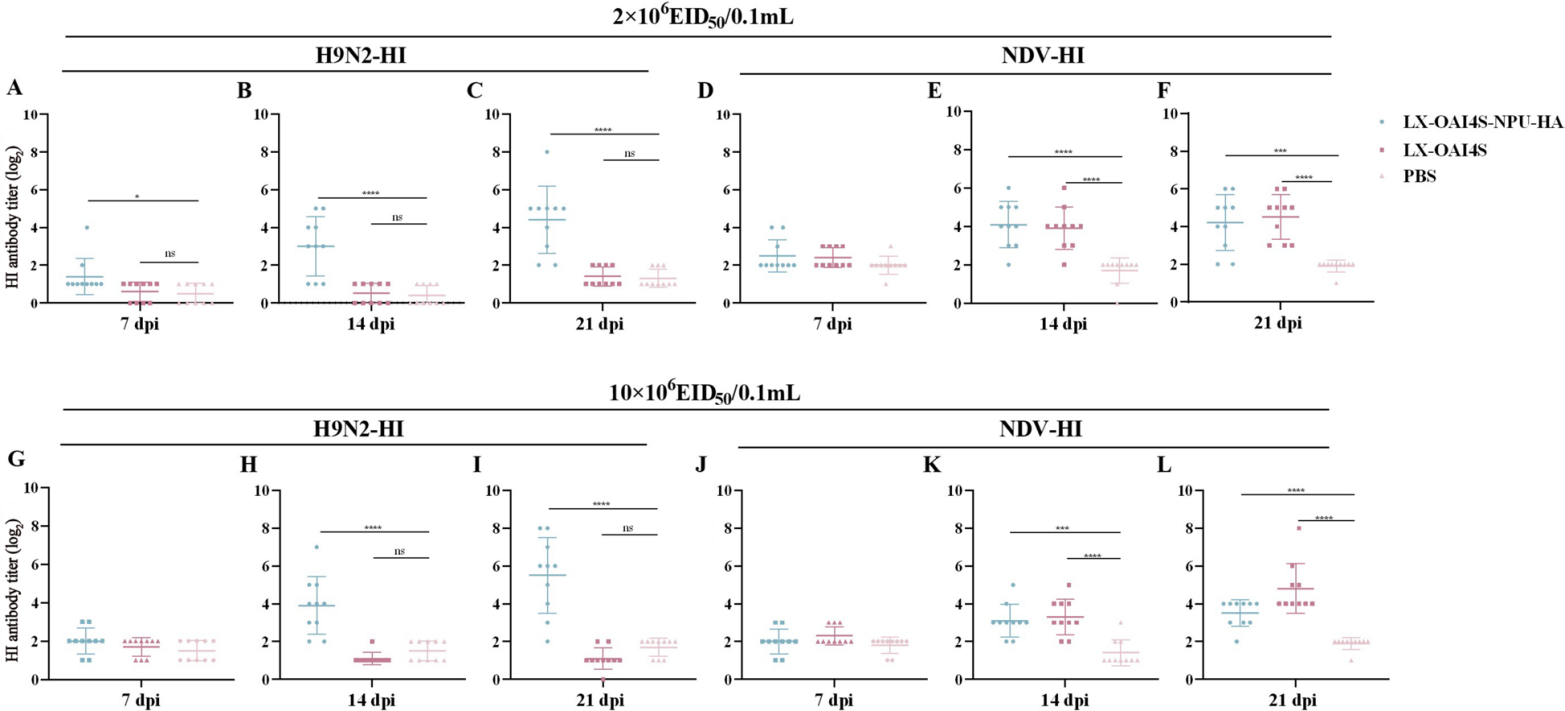
**HI antibody titers of 1-day-old SPF chickens immunized with different doses by spray route. A**–**C** HI (H9N2) antibody titre of PBS, LX-OAI4S-NPU-HA and LX-OAI4S immunized with 2 × 10^6^ EID_50_/0.1 mL. **D**–**F** HI (NDV) antibody titre of PBS, LX-OAI4S-NPU-HA and LX-OAI4S immunized with 2 × 10^6^ EID_50_/0.1mL. **G**–**I** HI (H9N2) antibody titre of PBS, LX-OAI4S-NPU-HA and LX-OAI4S immunized with 10 × 10^6^ EID_50_/0.1 mL. **J**–**L** HI (NDV) antibody titre of PBS, LX-OAI4S-NPU-HA and LX-OAI4S immunized with 10 × 10^6^ EID_50_/0.1 mL. The *P*-values shown for the comparison between PBS and LX-OAI4S-NPU-HA, as well as the rLX-OAI4S vaccine group. *P < 0.05; **P < 0.01; ***P < 0.001; ****P < 0.0001; ns, no significant difference

We conducted a 21-day surveillance period following spray immunisation to monitor the immunised chickens. Two chickens in the high-dose group (10 × 10^6^ EID_50_/0.1mL) showed slight respiratory symptoms after immunisation; however, this resolved within a few days. The remaining immunised groups did not present noteworthy respiratory symptoms. In the low-dose group (2 × 10^6^ EID_50_/0.1mL), the average value of HI antibody against H9N2 reached 4.4log_2_ at 21 dpi, and the average value of HI antibody against NDV reached 4.2log_2_. Both outcomes could, therefore, achieve the standard of immune protection produced by the vaccine [22]. In the high-dose group (10 × 10^6^ EID_50_/0.1 mL), the HI antibodies against H9N2 were approximately one titre higher (5.55log_2_) than those in the low-dose group at 21 dpi. In comparison, the HI antibodies against NDV were about one titre lower (3.36 log_2_) than those in the low-dose group (Figure 7).

Moreover, a significant outcome was noted at 7 dpc in both the high-dose and low-dose immunisation groups. Notably, there were no reports of respiratory or mental symptoms or deaths within the immunised groups post-challenge. The low-dose group effectively prevented the shedding of NDV, whereas it failed to inhibit the shedding of the H9N2 AIV. In contrast, the high-dose group could completely inhibit the virus shedding of NDV and H9N2 AIV (Tables 4 and 5).

**Table 4:** **Virus shedding of chickens immunized with different vaccine doses after H9N2 AIV challenge**

Groups	2 × 10^6^ EID_50_/0.1 mL		10 × 10^6^ EID_50_/0.1 mL	
	Oral	Cloacal	Oral	Cloacal
LX-OAI4S-NPU-HA	10/10	0/10	0/10	0/10
PBS	10/10	3/10	9/10	4/10

**Table 5:** **Virus shedding of chickens immunized with different doses of vaccine after NDV challenge**

Groups	2×10^6^ EID_50_/0.1 mL		10 ×10^6^ EID_50_/0.1 mL	
	Oral	Cloacal	Oral	Cloacal
LX-OAI4S-NPU-HA	1/10	1/10	0/10	0/10
LX-OAI4S	0/10	0/10	0/10	0/10
PBS	10/10	10/10	9/10	9/10

## Discussion

AI and ND are chronic threats to the poultry industry, and their prevention and control remain top priorities for the global industry [20]. H9N2 AIV acts as an ‘internal gene donor’ among various subtypes, escalating the complexity of AI prevention and control [21, 22]. Genotype VII NDV was previously the predominant genotype in China, inflicting substantial damage to the poultry industry [23]. Hence, developing vaccines that effectively prevent and control H9N2 AIV and genotype VII NDV is a paramount objective. Currently, commercially available inactivated bivalent or multivalent vaccines that target both diseases have been developed [24].

However, despite being scarce, live vaccines are unique in their ability to elicit humoral, mucosal, and cellular immune responses. Additionally, live vaccines enable versatile immunisation methods like spray applications, facilitating mass vaccination while conserving labour and resources [25]. Consequently, developing live vaccines for H9N2 AIV and NDV is imperative.

This study successfully created the recombinant virus based on the chimeric virus LX-OAI4S, a novel bivalent live vector vaccine against genotype VII NDV and H9N2 AIV. The recombinant virus retained the biological traits of its parent strain, and the insertion of exogenous genes did not significantly impact its virulence or replication capacity. The exogenous proteins were also effectively expressed, with variations observed across different chimeric constructs.

It has previously been demonstrated that NDV’s UTRs are key for viral replication and host pathogenicity [26]. Research also indicates that UTRs from different genes of the NDV have varying regulatory effects on the expression of exogenous proteins [18]. In this study, we inserted NDV gene UTRs around the H9N2 AIV HA gene, noting that the NP UTR significantly enhanced exogenous protein expression, with the HN UTR having a lesser effect. Recombinant viruses without NDV UTR fusion displayed lower exogenous gene expression than those with UTR fusion. This outcome shows that NDV UTRs flanking the exogenous gene boost protein expression, though the regulatory mechanisms need further investigation.

Additionally, immunisation of SPF chickens intranasally and ocularly led to increased HI antibody titres against NDV and H9N2. Furthermore, all three vaccine strains met the standard for an effective immune response in chickens [27]. The vaccine strain LX-OAI4S-NPU-HA exhibited a rapid and persistent production of antibodies. However, inactivated H9N2 vaccines often struggle to prevent virus shedding due to insufficient cellular and mucosal immunity [28, 29]. Live vector vaccines, conversely, trigger both humoral and cellular/mucosal immunity [30]. The study showed that the LX-OAI4S-NPU-HA vaccine, in particular, significantly reduced H9N2 and NDV virus shedding, with a 100% inhibition rate against virulent NDV. It also suppressed organ viral replication more than the control, especially against H9N2.

Conventional immunisation methods, such as intramuscular injection and nasal or ocular drops, are incompatible with modern breeding practices. Thus, vaccine development requires more convenient and practical administration routes [31]. Spray vaccination is commonly used in intensive farming for its efficiency and speed [32, 33]. NDV induces mucosal immunity in the upper respiratory tract; therefore, spray vaccination should be considered for mucosal immune response in young chicks to reduce early infection risk [34–36].

Here, we investigated the protective efficacy of LX-OAI4S-NPU-HA via spray vaccination, which induced adequate HI antibody titres (≥ 3log_2_). Our results showed no significant difference in HI antibody levels between high- and low-dose groups, aligning with the hypothesis that the effectiveness of vaccines plateaus at a certain titre threshold [37]. Our subsequent tests revealed that both the high- and low-dose vaccine groups fully protected chickens from NDV. The high-dose group also stopped H9N2 AIV from spreading. Therefore, administering LX-OAI4S-NPU-HA via a spray has a good safety profile and a low-stress response in chicks.

In summary, we successfully generated four recombinant NDVs expressing the H9 HA gene and evaluated the immunogenicity and protective efficacy against H9N2 AIV and NDV. Notably, the LX-OAI4S-NPU-HA strain exhibited the highest exogenous HA expression, significantly inducing HI antibodies against both viruses. Furthermore, NPUTR induced HI antibodies against H9N2 and NDV. It also offered 100% protection against NDV across high and low dosages and high protection against H9N2 AIV at high doses. Therefore, LX-OAI4S-NPU-HA emerges as a promising dual-use live vector vaccine candidate for H9N2 AIV and NDV.

## Supplementary Information

**Additional file 1**. **Primers designed for whole genome sequencing of rLX-OAI4S/Has.**

**Additional file 2**. **Primer sequence for RT-qPCR.**

**Additional file 3**. **Sequence alignment of HA and F genes after passage of recombinant virus.** Sequence alignment of HA genes and F genes by MEGA software. The red box represents the cleavage site of F genes. P1, P5, P10, P15, and P20 denote the 1st, 5th, 10th, 15th, and 20th sequential passages in chicken embryos, respectively.

## Data Availability

All data generated or analysed during this study are included in this published article.
