# Supplementary material for: Spray vaccination with a safe and bivalent H9N2 recombinant chimeric NDV vector vaccine elicits complete protection against NDV and H9N2 AIV challenge
Source: Vet Res. 2025 Jan 31;56:24. doi: 10.1186/s13567-025-01448-5 (PMC11786375; doi:10.1186/s13567-025-01448-5)
Supplement: Supplementary file 1 — Additional file 1. Primers designed for whole genome sequencing of rLX-OAI4S/Has. [file 13567_2025_1448_MOESM1_ESM.docx]

**Additional file 1 Primers designed for whole genome sequencing of rLX-OAI4S/HAs**

| Name | Sequence 5’-3’ |
| --- | --- |
| SacII-F | TCAACAACAGGGGAGTCAACAAAGCCGCGGAAACAGCCAAGAGAGACAC |
| P-NP5’UTR-R | **TCGCACTCGAGATTCACACC**TTCTACCCGTATTTTTTCTTAATCCTTAATATAGCTGATATAGCTGAAT |
| NP5’UTR-HA-F | **GGTGTGAATCTCGAGTGCGA**GCCCGAAGCTCAAACTCGAGAGAGCCTTCTGCCAAAATGGAGACAGTATCACTAATTTCTGCCAAAATGGAGACAGTATCACTAAT |
| HA-NP-R | **TTATATACAAATGTTGCATC**TGCAAGACCCATTGGA |
| HA-NP3’UTR-F | **GATGCAACATTTGTATATAA**CCGACAGCACCCAGT |
| NP3’UTR-R | **ATTCTACCCGTGATTTTTTCTTAATTTCTT**TGGCCCTG |
| NP3’UTR-M-F | **AAGAAATTAAGAAAAAATCACGGGTAGAAT**CGGAGTGCCCCGATTG |
| P-M5’UTR-R | **CTTGGCACAATCGGGGCACT**CCGATTCTACCCGTATTTTTTCTTAATCCTTAA |
| M5’UTR-HA-F | **AGTGCCCCGATTGTGCCAAG**ATGGAGACAGTATCACTAATAA |
| HA-M-R | **TTATATACAAATGTTGCATC**TGCAAGACCCATTGGACATGGCCCAGA |
| M3’TUR-F | **GATGCAACATTTGTATATAA**GCTGCATCTCTGAGAT TGC |
| M3’UTR-R | **TTCTACCCGTATTTTTTCTTAA**TTTGATAGAC |
| M3’UTR-M-F | **TTAAGAAAAAATACGGGTAGAA**TCGGAGTGCCCCGATGTGCCAAGATGGACT |
| P-HN5’UTR-R | **CCCGGCTCCCGATTGAGGGA**CGGCCTCCCCGACCGTTCTACCCGTCATTTTTTCTTAATCCTTAATATAGCT |
| HN5UTR-HA-F | **TCCCTCAATCGGGAGCCGGG**CCTCACAACATCCGTTCTACCGCATCACCAATAGCAGTTTTCAGTCATGGAGACAGTATCA |
| HA-HN3’UTR-R | **CAAGTGACTACCGACAAGACTTAAT**CTAATCACATTAGCACTAGCTGATTATATACAAATG |
| HN3’UTR-M-F | **ATTAAGTCTTGTCGGTAGTCACTTG**ATTAAGAAAAAATACGGGTAGAATCGGAGTGCCCCGATTGTG |
| P-HA-R | **CCATTTCTACCCGTATTTTTTCTTAA**TCCTTAATATAGCTGA |
| HA-F | **TTAAGAAAAAATACGGGTAGAAATGG**AGACAGTA |

Enzyme cleavage sites are marked with underscores, and homologous sequences are marked with bold.
