# Supplementary material for: Spray vaccination with a safe and bivalent H9N2 recombinant chimeric NDV vector vaccine elicits complete protection against NDV and H9N2 AIV challenge
Source: Vet Res. 2025 Jan 31;56:24. doi: 10.1186/s13567-025-01448-5 (PMC11786375; doi:10.1186/s13567-025-01448-5)
Supplement: Supplementary file 2 — Additional file 2. Primer sequence for RT-qPCR. [file 13567_2025_1448_MOESM2_ESM.docx]

**Additional file 2. Primer sequence for RT-qPCR**

| Name | Sequence 5’-3’ |
| --- | --- |
| H9-HA-PF | TATAATAAAGTGAAGAGGGCGTT |
| H9-HA-PR | TTCCGAATTGTCTCCATGC |
| H9-HA-P | AAAGGATGTTTCGAGCTATACCAC |
| LX-NP-PF | GCGCCGTACATGACATTG |
| LX-NP-PR | GAGCCTGAGCGTACTCTA |
| LX-NP-P | AGCACATCATTCTGGAGACTTGGA |
