# Supplementary figures and images for: Spray vaccination with a safe and bivalent H9N2 recombinant chimeric NDV vector vaccine elicits complete protection against NDV and H9N2 AIV challenge
Source: Vet Res. 2025 Jan 31;56:24. doi: 10.1186/s13567-025-01448-5 (PMC11786375; doi:10.1186/s13567-025-01448-5)

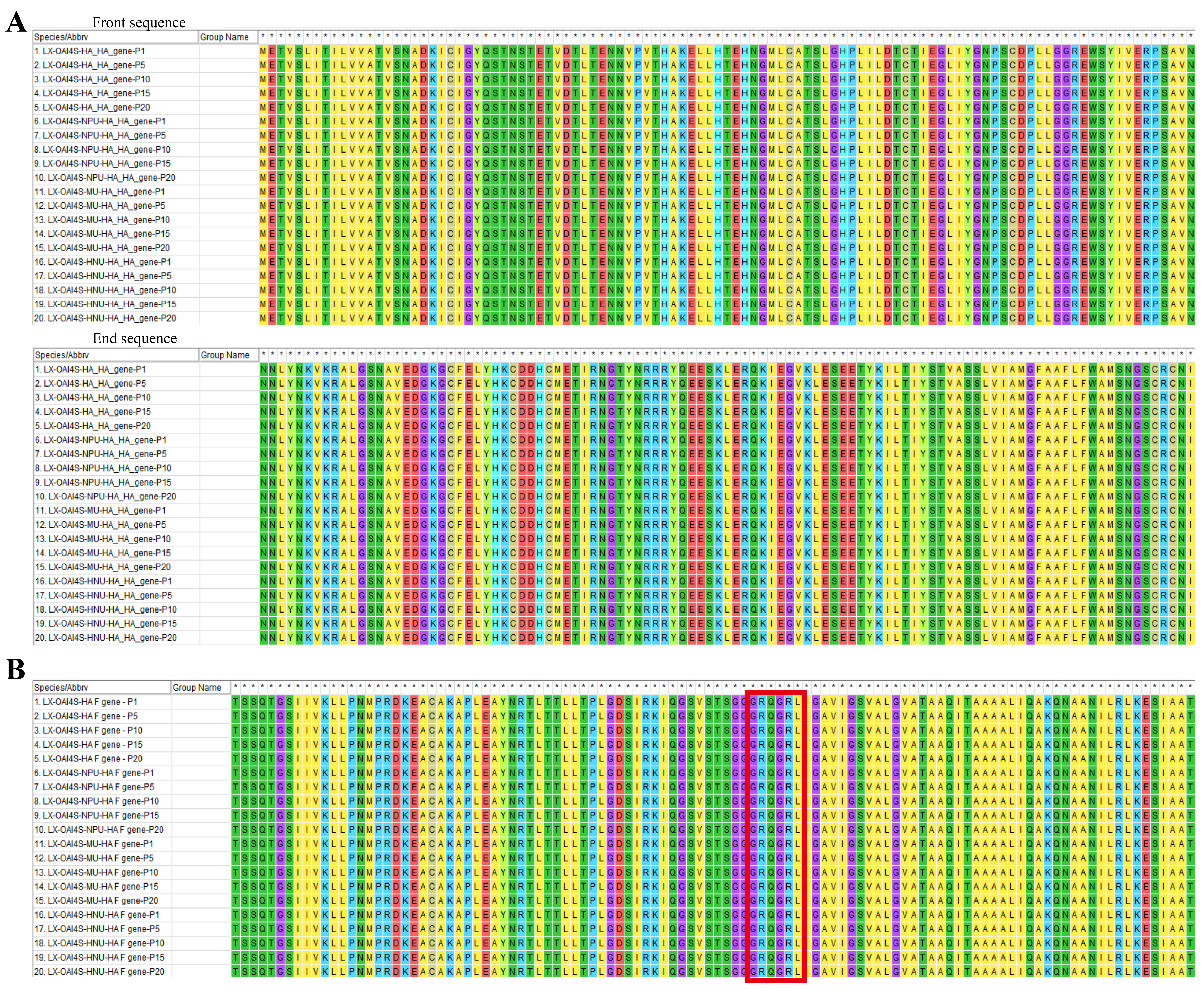

Supplement: Supplementary file 3 — Additional file 3. Sequence alignment of HA and F genes after passage of recombinant virus. Sequence alignment of HA genes and F genes by MEGA software. The red box represents the cleavage site of F genes. P1, P5, P10, P15, and P20 denote the 1st, 5th, 10th, 15th, and 20th sequential passages in chicken embryos, respectively. [file 13567_2025_1448_MOESM3_ESM.tif]
